# PB.31. Occult breast carcinoma: an experience at a single unit

**DOI:** 10.1186/bcr3732

**Published:** 2014-11-03

**Authors:** S Meraj, C Low, V Gaur, S Muthyala

**Affiliations:** 1 UHCW NHS Trust, Coventry, UK

## Introduction

The breast carcinoma presents rarely as metastases to the axillary nodes without an obvious breast lesion seen on conventional imaging such as mammography and US. In this study we looked at the usefulness of DCE-MRI of the breast in identifying these tumours at a single institute. In addition we evaluated the use of second-look US and MRI-guided biopsy.

## Methods

A total of 10 patients were identified from our local MR database who were diagnosed with occult breast cancer between February 2012 and April 2014. The data were collected from PACS and the hospital RIS.

## Results

All patients presented with axilllary nodes and had a normal clinical examination of the breast and had normal mammograms. The majority of these patients (80%) had mixed-density breast parenchyma. In 80% of patients the cancer could be diagnosed on the DCE-MRI of the breast. The remaining two patients had normal MR examination. The morphological features of the lesions were nonmass-like enhancement, spiculate and lobulated masses, ranging in size from 6 to 35 mm. Fifty per cent of these patients demonstrated type 3 kinetic curves. In 62.5% of patients with positive MR, the lesion could be identified and biopsied on the second-look US. In the remaining two cases the tumour was diagnosed with MRI-guided biopsy. Most patients underwent wire-guided WLE and ANC. The majority of these cancers (75%) were ductal NST.

## Conclusion

Our experience is similar to previously published literature in that MRI aids in identifying most of the occult cancers.

